# Deep Sequencing Analysis of miRNA Expression in Breast Muscle of Fast-Growing and Slow-Growing Broilers

**DOI:** 10.3390/ijms160716242

**Published:** 2015-07-17

**Authors:** Hongjia Ouyang, Xiaomei He, Guihuan Li, Haiping Xu, Xinzheng Jia, Qinghua Nie, Xiquan Zhang

**Affiliations:** 1 Department of Animal Genetics, Breeding and Reproduction, College of Animal Science, South China Agricultural University, Guangzhou 510642, China; E-Mails: oyolive@stu.scau.edu.cn (H.O.); 13533993199@163.com (X.H.); yunturn@126.com (G.L.); music-three@163.com (H.X.); xinzhengjia@126.com (X.J.); xqzhang@scau.edu.cn (X.Z.); 2 Guangdong Provincial Key Lab of Agro-Animal Genomics and Molecular Breeding and Key Lab of Chicken Genetics, Breeding and Reproduction, Ministry of Agriculture, Guangzhou 510642, China

**Keywords:** chicken, miRNA, high throughput sequencing, growth

## Abstract

Growth performance is an important economic trait in chicken. MicroRNAs (miRNAs) have been shown to play important roles in various biological processes, but their functions in chicken growth are not yet clear. To investigate the function of miRNAs in chicken growth, breast muscle tissues of the two-tail samples (highest and lowest body weight) from Recessive White Rock (WRR) and Xinghua Chickens (XH) were performed on high throughput small RNA deep sequencing. In this study, a total of 921 miRNAs were identified, including 733 known mature miRNAs and 188 novel miRNAs. There were 200, 279, 257 and 297 differentially expressed miRNAs in the comparisons of WRRh *vs.* WRRl, WRRh *vs.* XHh, WRRl *vs.* XHl, and XHh *vs*. XHl group, respectively. A total of 22 highly differentially expressed miRNAs (fold change > 2 or < 0.5; *p*-value < 0.05; *q*-value < 0.01), which also have abundant expression (read counts > 1000) were found in our comparisons. As far as two analyses (WRRh *vs.* WRRl, and XHh *vs.* XHl) are concerned, we found 80 common differentially expressed miRNAs, while 110 miRNAs were found in WRRh *vs.* XHh and WRRl *vs.* XHl. Furthermore, 26 common miRNAs were identified among all four comparisons. Four differentially expressed miRNAs (miR-223, miR-16, miR-205a and miR-222b-5p) were validated by quantitative real-time RT-PCR (qRT-PCR). Regulatory networks of interactions among miRNAs and their targets were constructed using integrative miRNA target-prediction and network-analysis. Growth hormone receptor (GHR) was confirmed as a target of miR-146b-3p by dual-luciferase assay and qPCR, indicating that miR-34c, miR-223, miR-146b-3p, miR-21 and miR-205a are key growth-related target genes in the network. These miRNAs are proposed as candidate miRNAs for future studies concerning miRNA-target function on regulation of chicken growth.

## 1. Introduction

Chicken growth traits play a crucial role in poultry production, which is affected by multiple factors, including genetic, nutritional, and environmental features [1]. In the past two decades, many growth candidate genes and quantitative trait loci (QTLs) for chicken growth have been identified [2,3,4,5,6,7]. With the development of next generation sequencing, a number of genome-wide association studies (GWAS) and transcriptome sequencing analyses were performed; more candidate genes and significant SNPs were found to influence chicken growth traits [8,9,10,11,12]. However, there are few studies about other genetic factors affecting chicken growth, such as microRNA regulation and epigenetic inheritance, and the genetic mechanisms of chicken growth are far from clear.

MicroRNAs, a class of non-coding small RNA with 20–23 nucleotides in length, can regulate gene expression by targeting specific sites in the 3′-untranslated region (3′-UTR) of mRNAs [13]. Increasing studies indicate that miRNAs widely control biological and metabolic processes by regulating post-transcriptional gene expression [14,15,16]. Furthermore, variations of microRNA may lead to abnormal function, and change the phenotypic character [17,18]. Until now, chicken has the highest numbers of identified miRNAs in domestic animals; 1058 chicken miRNAs have been identified based on the database of miRase 21.0 [19]. Several studies have found miRNAs associated with embryo development [20,21], cell proliferation [22], immunity function [23,24,25], and skeletal muscle development in chicken [26]. Therefore, miRNAs may play an important role in affecting chicken growth traits.

The aim of the present study was to identify miRNAs associated with growth traits in chicken. Breast muscle tissues of two-tail samples from WRR and XH, which exhibit different growth performance at seven weeks of age, were used for high throughput small RNA deep sequencing to detect the differentially expressed miRNAs.

## 2. Results

### 2.1. Overview of Small RNA Deep Sequencing Data

**Figure 1:**
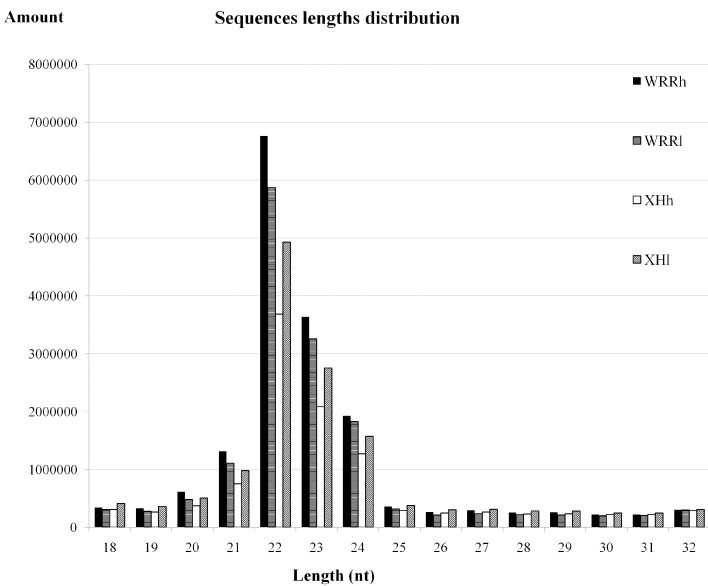
Length distribution and abundance of small RNAs sequences in chicken breast tissue. Clean reads of 18–32 nt for all four groups were assessed for size distribution; and the most abundant size class was 22 nt; followed by 23 and 24 nt.

In this study, three pooled breast muscle tissues for each group of WRRh (the group of Recessive White Rock with high body weight), WRRl (the group of Recessive White Rock with low body weight), XHh (the group of Xinhua Chickens with high body weight) and XHl (the group of Xinhua Chickens with low body weight) were sequenced simultaneously by Illumina Solexa sequencing. All sequencing data were submitted to the NCBI GEO database with the accession number GSE62971 [27]. A range of 22,080,436 to 16,410,221 raw reads for the four groups was obtained. Firstly, low-quality reads and meaningless reads were filtered out. The quality data for RNA samples are shown in Figure S1. A total of 20,424,161, 18,160,137, 14,806,039 and 17,295,270 clean reads was obtained for WRRh, WRRl, XHh and XHl, respectively (Table 1). The size distribution of clean reads was assessed for all four groups. Small RNA sequence length was mainly concentrated at 21–24 nt, and the length of 22 nt was the maximum size (Figure 1).

**Table 1:** Summary of data generated from small RNA deep sequencing.

Sample	Total Reads	Clean Reads	Unique Reads	Mapped Reads ^a^	Percentage ^b^
**WRRh**	22,080,436	20,424,161	577,820	433,846	75.08%
**WRRl**	19,917,025	18,160,137	513,726	392,323	76.37%
**XHh**	16,410,221	14,806,039	494,539	366,898	74.19%
**XHl**	19,473,651	17,295,270	625,063	482,989	77.27%

^a^ The unique reads mapped in chicken genome; ^b^ The percentage of mapped reads in unique reads.

**Figure 2:**
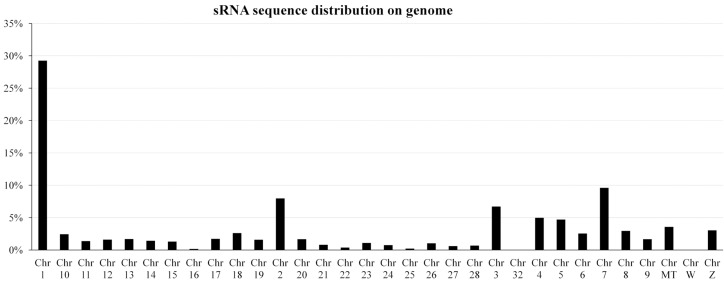
Distribution percentages of unique small RNAs sequences on chicken chromosome. The unique small RNA reads were mapped to chromosome by BLASTing with the chicken genome; and then the percentages of mapped reads of each chromosome in total mapped reads were then calculated. Chr MT: mitochondrial genome.

**Figure 3:**
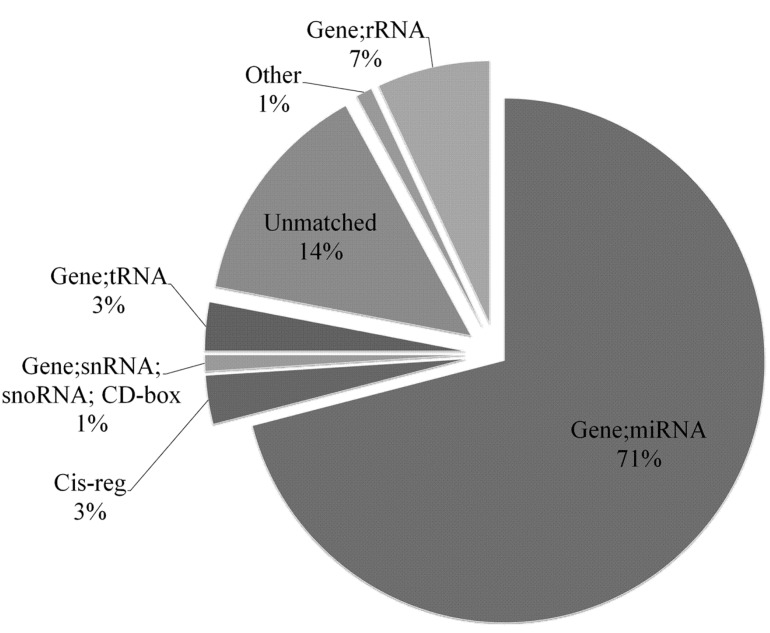
Frequency of unique small RNA distribution among the different categories. The unique sequences were subjected to searches for the types and numbers of sRNA using the Rfam databases (rRNA; tRNA; sn/snoRNA; miRNAs; other noncoding RNA).

Then, the clean reads were assembled by groups, giving rise to 577,820, 513,726, 494,539, and 625,063 unique sequences for WRRh, WRRl, XHh, and XHl, respectively (Table 1). The unique small RNA reads were mapped to the chromosome by blasting with the chicken genome. Results showed that over 70% of the reads could be perfectly mapped to the chicken genome. Moreover, they were mainly located at chromosome 1 (29.25%), 2 (7.96%), 3 (6.71%), 4 (4.97%), 5 (4.71%) and 7 (9.59%) (Figure 2). Finally, the type and number of sRNA were searched using Rfam databases (rRNA, tRNA, sn/snoRNA, miRNAs, other noncoding RNA). The unique sequences were categorized into seven groups; 71.0% of them were defined as miRNAs, 14% were unmatched, and 15% were other known categories of identified small RNA including rRNA, tRNA, snRNA, snoRNA, *etc.* (Figure 3).

### 2.2. Characterization of Known miRNAs and Predicted Novel miRNAs

Known conserved miRNAs were identified from the breast muscle of four groups using miRBase 21.0 (http://www.mirbase.org/), and novel miRNAs were also predicted from unannotated sRNA by miRDeep2 [28]. In this study, a total of 921 miRNAs (733 known mature miRNAs and 188 novel miRNAs) were detected. There were 891 and 797 miRNAs in WRR and the XH chicken each, of which 477 known mature miRNAs were expressed in all four groups (Table S1 in supplementary materials).

The abundance of the miRNAs could reflect differences in the roles of these miRNAs in the regulation of growth. The top 20 abundant known miRNAs in our libraries are listed in Table 2. The most abundant miRNAs is the gga-miR-133 family, which includes gga-miR-133a, gga-miR-133b and gga-miR-133c. The let-7 family was also expressed abundantly in the breast muscle libraries, five of them (gga-let-7a, gga-let-7c, gga-let-7f, gga-let-7j and gga-let-7k) are in the list of the top 20 abundant miRNAs. We found that putative novel miRNAs were less abundant than known miRNAs. There were 12 putative novel miRNAs and 131 known miRNAs in the libraries which have read counts greater than 1000 (Table S2 in supplementary materials). The predicted secondary structures of the two most abundant novel miRNAs are shown in Figure S2. Comparing the chicken miRNAs sequences, heterogeneity at the 5′ and/or 3′ ends of miRNAs was observed (Figure S3). These variations of miRNAs from their miRBase reference sequences are referred to as isomiRs [29]. We found that many miRNAs have various isoforms in chicken breast muscle libraries, and some miRNAs have more than one highly abundant isoform (e.g., gga-let-7c, gga-miR-205a and gga-miR-223). In addition to a few miRNAs (e.g., gga-let-7c), the most abundant isoforms are identical to the reference in miRBase. The most highly expressed miRNAs appear to have a greater number of different isoforms, e.g., 26 isoforms of gga-let-7c were identified.

**Table 2:** The top 20 abundant known miRNAs in chicken breast muscles.

miRNAs	Normalized Reads				Total Reads
	WRRh	WRRl	XHh	XHl	
**gga-miR-133a**	3,558,683	3,069,071	1,997,286	2,607,787	11,232,827
**gga-miR-133c**	3,350,936	2,885,440	1,878,925	2,449,209	10,564,510
**gga-miR-133b**	3,326,848	2,864,578	1,864,721	2,431,274	10,487,421
**gga-let-7a**	1,699,621	1,513,865	857,210	1,133,532	5,204,228
**gga-miR-22-3p**	1,333,233	1,145,421	712,464	988,186	4,179,304
**gga-miR-30a-5p**	1,213,468	1,148,128	790,893	930,507	4,082,996
**gga-miR-26a**	1,212,635	1,054,689	691,456	1,006,522	3,965,302
**gga-miR-30d**	851,887	813,262	583,932	667,002	2,916,083
**gga-miR-181a-6p**	918,452	836,452	485,661	650,836	2,891,401
**gga-miR-10a-5p**	943,686	782,180	420,809	663,401	2,810,076
**gga-miR-10b**	911,725	757,564	398,852	633,567	2,701,708
**gga-miR-30e**	799,679	730,832	501,718	596,218	2,628,447
**gga-let-7j**	848,972	756,205	428,182	566,165	2,599,524
**gga-let-7f**	398,292	363,598	206,995	274,333	1,243,218
**gga-miR-148a**	288,585	300,432	144,015	180,973	914,005
**gga-miR-146c-5p**	224,147	207,782	171,443	132,712	736,084
**gga-let-7k**	211,853	206,518	118,297	155,412	692,080
**gga-let-7c**	242,661	189,820	111,118	139,257	682,856
**gga-miR-199-3p**	168,417	152,158	75,346	121,460	517,381
**gga-miR-126-5p**	139,914	109,805	89,607	86,681	426,007

### 2.3. Identification of Differentially Expressed miRNAs

Differentially expressed miRNAs were identified by DEGseq analysis (fold change > 1.5 or < 0.66; *p*-value < 0.05; *q*-value < 0.01), as a result, 200, 279, 257 and 297 miRNAs were detected in four comparisons of WRRh *vs.* WRRl, WRRh *vs.* XHh, WRRl *vs.* XHl and XHh *vs.* XHl, respectively. Multiple comparisons revealed 80 miRNAs within breeds (WRRh *vs.* WRRl and XHh *vs.* XHl), and 110 miRNAs between breeds (WRRh *vs.* XHh and WRRl *vs.* XHl). The details of differentially expressed miRNAs are shown in Table S3 in supplementary materials. Among them, moreover, 26 miRNAs (including 10 known miRNAs of miR-122, miR-1329-3p, miR-1587, miR-1736-3p, miR-1769-3p, miR-1769-5p, miR-1773-5p, miR-205a, miR-31 and miR-375) were found in all four comparisons (Table 3). Furthermore, we focused on the miRNAs that were both abundant (read counts > 1000) and highly differentially expressed (fold change > 2 or < 0.5; *p*-value < 0.05; *q*-value < 0.01) in our comparisons, and found that 22 miRNAs met the standards (Table 4).

**Table 3:** Statistics of significant differently expressed miRNAs.

Sample 1 *vs.* Sample 2	Up ^a^	Down ^b^	Total	Shared	
**WRRh *vs.* WRRl**	116 (47)	84 (24)	200 (71)	80 (22)	26 (16)
**XHh *vs.* XHl**	129 (44)	150 (40)	279 (84)		
**WRRh *vs.* XHh**	130 (31)	127 (47)	257 (78)	110 (39)	
**WRR *vs.* XHl**	137 (41)	160 (60)	297 (101)		

^a^ The numbers of miRNAs expressed in sample 1 more than sample 2; ^b^ The numbers of miRNAs expressed in sample 1 were lower than sample 2. The value in brackets is the number of novel miRNAs. WRRh *vs.* WRRl indicated the comparison between the two-tail samples of Recessive White Rock; XHh *vs.* XHl indicated the comparison between the two-tail samples of Xinhua Chickens; WRRh *vs* XHh indicated the comparison between the groups of Recessive White Rock and Xinhua Chickens with high body weight; WRRl *vs.* XHl indicated the comparison between the groups of Recessive White Rock and Xinhua Chickens with low body weight.

**Table 4:** Differently expressed miRNAs and their candidate target genes.

miRNAs	Normalized Reads		Fold Change	Target Gene
	Sample 1	Sample 2		
**gga-miR-1329-5p**	WRRh 459	WRRl 1582	0.29	*EDNRB*; *WWP2*
	WRRl 1582	XHl 174	9.07	
**gga-miR-1416-5p**	XHh 5680	XHl 2721	2.09	*CTGF*; *CXCL12*
	WRRl 8061	XHl 2721	2.96	
**gga-miR-142-5p**	WRRh 108,463	XHh 54,059	2.01	*FOXO3*; *ITGA8*
	WRRl 173,442	XHl 71,860	2.41	
**gga-miR-146b-3p**	WRRl 5800	XHl 2404	2.41	*TNNC1*; *AKT1*; *GHR*
**gga-miR-146b-5p**	WRRl 191,004	XHl 71,169	2.68	–
**gga-miR-147**	WRRh 882	WRRl 2824	0.31	–
	WRRl 2824	XHl 928	3.04	
**gga-miR-155**	WRRh 5205	WRRl 14,058	0.37	*ACTA1*; *AKT1*; *EDNRA*
	WRRh 5205	XHh 1759	2.96	
	WRRl 14,058	XHl 2018	6.97	
**gga-miR-1744-3p**	WRRh 2359	WRRl 740	3.19	*TMOD1*; *EDN3*
**gga-miR-1769-3p**	WRRh 377	WRRl 1120	0.34	*DMD*; *BMP10*
	WRRl 1120	XHl 498	2.25	
**gga-miR-184**	WRRh 382	XHh 1735	0.22	*COL11A1*
**gga-miR-194**	WRRh 946	WRRl 4669	0.20	*MYBPC3*; *GCG*
	WRRh 946	XHh 1981	0.48	
	WRRl 4669	XHl 1470	3.18	
**gga-miR-204**	XHh 2762	XHl 5848	0.47	*MYO6*; *PDGFRA*; *SOCS3*
**gga-miR-205a**	WRRh 4900	WRRl 2281	2.15	*CISH*; *IFNG*
	WRRh 4900	XHh 2137	2.29	
	WRRl 2281	XHl 940	2.43	
**gga-miR-21**	WRRh 489,673	WRRl 1,060,669	0.46	*CISH*
	WRRl 1,060,669	XHl 360,267	2.94	
**gga-miR-222b-3p**	WRRh 2477	WRRl 6641	0.37	*ITGB1*
	WRRh 2477	XHh 658	3.77	
	WRRl 6641	XHl 791	8.40	
**gga-miR-223**	WRRl 10,225	XHl 4004	2.55	*MYH10*; *ADAM17*; *FOXO3*
**gga-miR-34b**	XHh 41	XHl 1750	0.02	*MYH10*; *BMP10*
	WRRl 218	XHl 1750	0.12	*STRAP*; *THY1*
**gga-miR-34c**	XHh 41	XHl 1750	0.02	*MYH7B*; *APP*
	WRRl 218	XHl 1750	0.12	*THY1*; *COL11A1*; *SOCS3*; *GCG*
**gga-miR-383**	WRRh 373	1603	0.23	*MYH7*; *APP*; *ITGA8*
**gga-miR-499**	WRRl 1754	XHl 6084	0.29	*ADAM17*; *FLNB*
**gga-miR-9-3p**	XHh 82	XHl 1177	0.07	*ACTG1*
	WRRl 81	XHl 1177	0.07	
**gga-miR-9-5p**	XHh 1036	XHl 3095	0.33	*TGFB2*

The miRNAs were both abundant (read counts > 1000) and highly differentially expressed (fold change > 2 or < 0.5; *p*-value < 0.05; *q*-value < 0.01) in our comparisons; the target genes were involved in growth related gene interaction networks.

**Figure 4:**
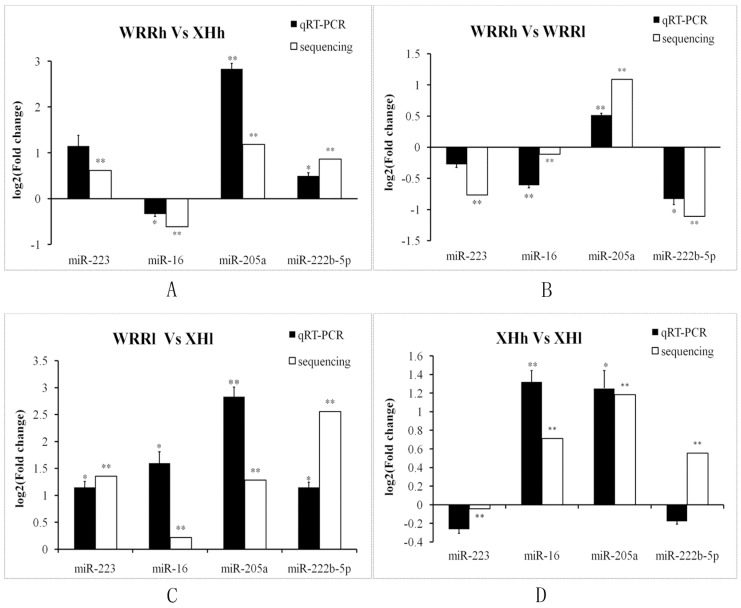
qRT-PCR validation of four differentially expressed miRNAs in all four comparisons. (**A**) WRRh *vs*. XHh; (**B**) WRRh *vs.* WRRl; (**C**) WRRl *vs.* XHl; (**D**) XHh *vs.* XHl. qRT-PCR reactions were run in triplicates and presented as means ± S.E.M. The Student’s *t*-test was used to compare expression levels among different groups. * *p* < 0.05; ** *p* < 0.01. WRRh *vs.* WRRl indicated the comparison between the two-tail samples of Recessive White Rock; XHh *vs.* XHl indicated the comparison between the two-tail samples of Xinhua Chickens; WRRh *vs.* XHh indicated the comparison between the groups of Recessive White Rock and Xinhua Chickens with high body weight; WRRl *vs.* XHl indicated the comparison between the groups of Recessive White Rock and Xinhua Chickens with low body weight.

To verify the RNA-Seq data, the differential expression of four miRNAs including miR-223, miR-16, miR-205a and miR-222b-5p were validated by qRT-PCR among all four comparisons (Figure 4). In general, except for miR-222b-5p in XHh *vs.* XHl contrast, the expression patterns of these four miRNAs were consistent with the RNA-Seq results, indicating that the deep sequencing results were reliable and appropriate for further analysis.

### 2.4. Target Gene Prediction, Function Annotation and Network Analysis

Through miRanda and RANhybrid, a total of 22,194 consensus potential miRNAs targets were obtained for all differentially expressed miRNAs. For all potential targets, GO annotation and KEGG pathway analysis were performed to identify functional modules. The GO results showed that 16,428 genes were involved in the categories and 502 biological process categories were significantly enriched (*p* < 0.05). The top 29 GO terms with enriched genes over 1000 were listed in Table S4 in supplementary materials. Pathway analysis of all targets revealed that 65 KEGG pathways were significantly enriched (*p* < 0.05), including 13 in WRRh *vs.* WRRl, 12 in XHh *vs.* XHl, 8 in WRRh *vs.* XHh, and 15 in WRRl *vs.* XHl (Table S5 in supplementary materials). Lysosome and metabolic pathways were enriched in all four comparisons.

**Figure 5:**
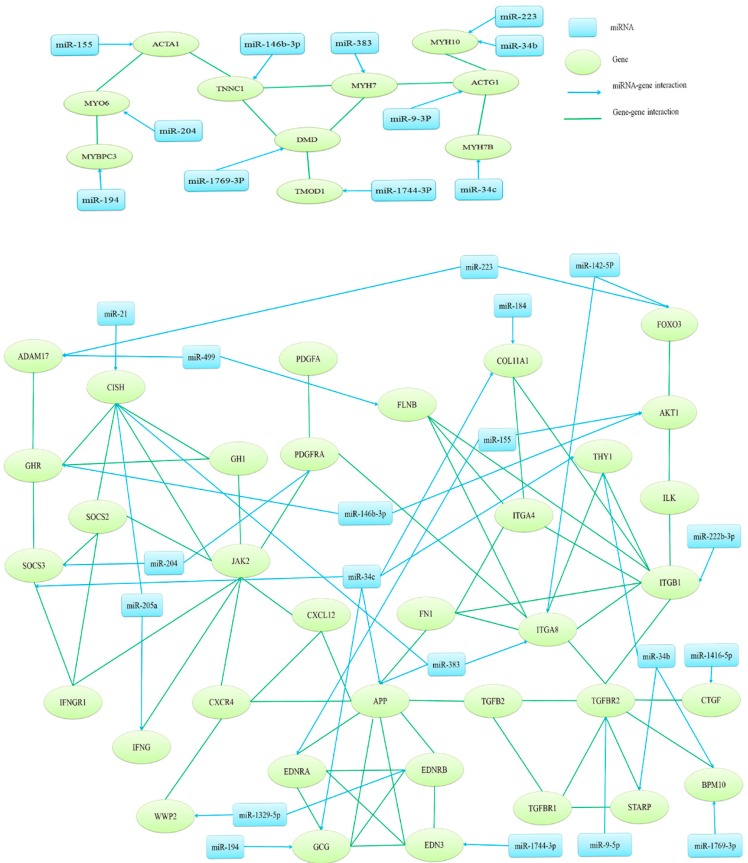
Interaction network of highly differentially expressed miRNAs and their potential targets. Candidate miRNA targets were obtained from growth related GO terms; and target–target pair interactions were searched using the STRING database. In the network; blue nodes denoted miRNAs and blue arrows denoted miRNA-target interaction; green nodes denoted targets and green lines denote target–target interaction.

For the 22 highly differentially expressed miRNAs (Table 4), 3151 potential targets were obtained by both miRanda [30] and RNAhybrid [31]. The further GO analysis for these targets showed that 192 biological process categories were significantly enriched (*p* < 0.05). The important growth-related GO terms are listed in Table 5, and are involved in regulation of growth, cell growth, muscle cell differentiation and development, and the transforming growth factor beta receptor signaling pathway. These GO terms contained 87 target genes, and their interactions were predicted using database of STRING 9.1 [32]. Two possible regulatory networks of interactions among miRNAs and their targets were constructed (Figure 5). A total of 20 miRNAs and 34 targets (Table 4) were involved in the two networks presented. Most of these 20 miRNAs have rarely been identified for their functional role in growth or muscle development. However, a number of their target genes have been reported to play a key role in regulation of growth, such as *GHR*, *CISH*, *SOCS3*, *APP*, *TGFBR2*, *AKT1* and *FOXO3*, *etc*. [33]. These miRNAs could participate in the regulation of growth through their target genes. For instance, gga-miR-34c has six targets in the present network, including *SOCS3*, *GCG*, *APP*, *THY1*, *COL11A1* and *MYH7B*; gga-miR-146b-3p was predicted to target *GHR* and *AKT1*; gga-miR-223 was predicted to target *FOXO3* and *ADAM17*; and the predicted target gene of gga-miR-9-5p was *TGFBR2*.

**Table 5:** Growth related biological processes identified by GO analysis for target genes of 22 highly differentially expressed miRNAs.

GO Accession	GO Terms	Gene Numbers	Fold Enrichment	*p* Value
GO:0040008	regulation of growth	29	1.5	0.016
GO:0001558	regulation of cell growth	16	1.8	0.019
GO:0045927	positive regulation of growth	11	2	0.029
GO:0030307	positive regulation of cell growth	5	3.1	0.025
GO:0017015	regulation of transforming growth factor β receptor signaling pathway	5	2.8	0.034
GO:0055001	muscle cell development	11	2.4	0.034
GO:0051146	striated muscle cell differentiation	13	1.8	0.040
GO:0060415	muscle tissue morphogenesis	6	2.7	0.049
GO:0003012	muscle system process	13	1.8	0.049
GO:0060537	muscle tissue development	17	1.6	0.036
GO:0055002	striated muscle cell development	8	2.1	0.035
GO:0014706	striated muscle tissue development	16	1.5	0.027

### 2.5. Validation of miR-146b-3p Targeted GHR Gene

**Figure 6:**
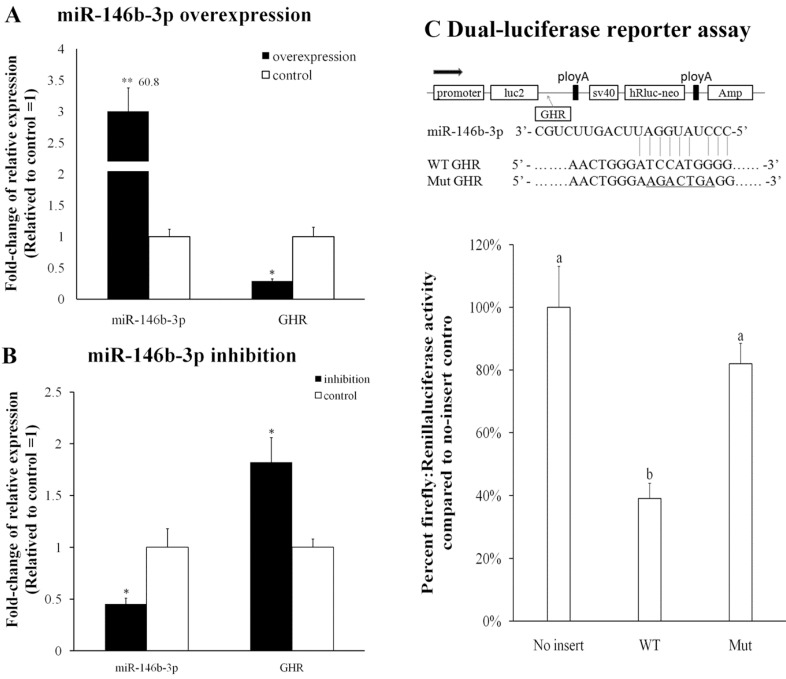
*GHR* is a target of miR-146b-3p in chickens. (**A**) The mRNA expression of *c-GHR* is significantly reduced after miR-146b-3p transfection in DF-1; (**B**) The mRNA expression of *c-GHR* is significantly increased after miR-146b-3p inhibitor transfection in DF-1; (**C**) DF-1 cells were transfected with miR-146b-3p mimic and co-transfected with no-insert control; *c-GHR* wild-type or mutant luciferase reporters. The predicted binding site and mutated site of miR-146b-3p in *c-GHR* is shown. All of the results are expressed as the mean ± S.E.M. of three replicates. * *p* < 0.05; ** *p* < 0.01; ^a,b^ *p* < 0.05. WT indicates *c-GHR* wild-type luciferase reporter and Mut indicates *c-GHR* mutant luciferase reporter.

**Figure 7:**
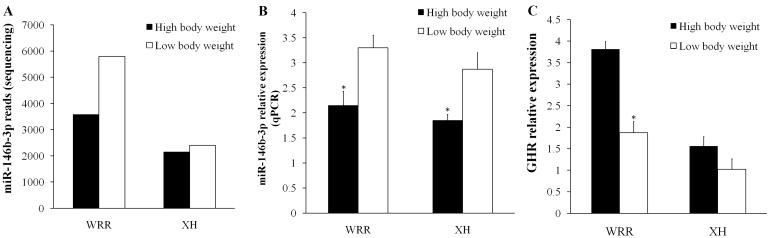
The expression of miR-146b-3p and GHR in breast muscle of two-tail samples of WRR and XH. (**A**) The reads of miR-146b-3p in four sequencing samples; (**B**) The expression of miR-146b-3p in two-tail samples of WRR and XH detected by qPCR; (**C**) The expression of *GHR* in two-tail samples of WRR and XH detected by qPCR. All of the results are expressed as the mean ± S.E.M. of three replicates. * *p* < 0.05.

DF-1 cells were transfected with miR-146b-3p mimics or inhibitor, and then the expression levels of miR-146b-3p and the *GHR* gene were detected after 48 h. The results show that overexpression of miR-146b-3p downregulated *GHR* mRNA expression (Figure 6A), and that the inhibition of endogenous miR-146b-3p increased *GHR* mRNA expression (Figure 6B). We constructed two dual-luciferase reporters with the wild-type or mutant of *GHR* inserted at the 3′ end of the firefly luciferase gene (Figure 6C). Dual-luciferase reporters assay showed that miR-146b-3p significantly reduced the firefly luciferase activity of the wild-type GHR reporter (*p* < 0.05) compared with no-insert control (Figure 6C). Furthermore, when miR-146b-3p was co-transfected with the mutant reporter, the firefly luciferase activity was only slightly decreased (*p* > 0.05) compared with the no-insert control. The expression of miR-146b-3p and *GHR* were detected in our sequencing samples, and the results indicate that their expression reveals a negative relationship (Figure 7). The expression of miR-146b-3p was higher (*p* < 0.05) in chickens with low body weight than those with high body weight in both WRR and XH chickens (Figure 7B), while the expression of *GHR* was higher in chickens with high body weight than those with low body weight (Figure 7C). These results indicated that the predicted site of *GHR* is a target of miR-146b-3p.

## 3. Discussion

Small RNA sequencing can uncover miRNAs expression at an overall level; it has become a useful tool for identifying functional miRNAs. Many miRNAs have been identified as being associated with animal growth performance by high-throughput sequencing [26,34,35]. In this study, we firstly reported the miRNAs expression profiles of chicken breast muscle between fast-growing and slow-growing chicken breeds. The sequence analysis showed that the main size of small RNAs in chicken breast muscle was 21–24, and 22 nt is the predominant size. This result was consistent with the known 19–24 nt range for miRNAs, and previous studies in chicken skeletal muscle and ovary also have similar findings [36,37]. In our sequencing libraries, a total of 921 miRNAs were detected, including 733 known miRNAs and 188 novel miRNAs. The three miRNAs of the gga-miR-133 family (gga-miR-133a, gga-miR-133b and gga-miR-133c) were the most abundant miRNAs in our breast muscle libraries. Previous studies showed that miR-133a was specifically expressed in muscle and regulates the process of skeletal muscle proliferation [38]. Overexpression of miR-133 could inhibit myoblast differentiation, but promotes myoblast proliferation by targeting *SRF* (serum response factor) [39]. The other abundantly expressed miRNAs family in our libraries was gga-let-7, which also was reported to have abundant expression in chicken skeletal muscle [40]. The family of let-7 miRNAs has been shown to play vital roles in mediating cell proliferation and differentiation, in particular, gga-let-7b has demonstrated a role in growth regulation through targeting *GHR* [26]. Of the top 20 abundantly expressed miRNAs identified, some, such as miR-133, miR-10b, miR-26a, miR-30e and gga-miR-30a were also abundantly expressed in chicken somites [41].

miRNAs have an important role in muscle tissues during embryonic development and miR-133, miR-1 and miR-206 are crucial regulatory factors during myogenesis [19,38,42,43]. In the present study, gga-miR-133 was expressed most highly in our samples, which indicated that it might also play a key role in chicken postnatal stages. Furthermore, miR-206 was also abundantly expressed in our samples, while miR-1 was not. These results suggest that some miRNAs might regulate muscle tissues during all development process, whereas some might play a key role only during a special muscle development stage. Differentially expressed miRNAs were confirmed by qPCR analysis. The good correlation between the two methods indicated that deep sequencing results were credible. A total of 22 highly differentially expressed miRNAs (fold change > 2 or < 0.5; *p*-value < 0.05; *q*-value < 0.01), which also have abundant expression (read counts > 1000) were found in our comparisons. Among these miRNAs, miR-21 was found to be highly expressed in many species, and associated with cardiac disease and a wide variety of human cancers [44,45]. In this study, miR-21 was upregulated in low-body weight of both WRR and XH chickens. Different expression levels of miR-21 were found in chicken skeletal muscle of different growth periods [26], and in skeletal muscle between broiler and layer [37]. One recent study showed that miR-21 can inhibit cell proliferation [46], suggesting that it might be a negative regulatory factor for chicken growth. These highly differentially expressed miRNAs may play a key role in chicken growth traits, and could be used as candidate genes for further study. However, with the exception of miR-21, most of these miRNAs were little known in terms of growth. It is known that the effects of miRNAs are mainly through regulating the expression of target genes [13]. Therefore, target gene prediction, and annotation of their biological function is useful to predict miRNA function. In this study, we used two different methods to predict the targets for the differently expressed miRNAs, and removed the different targets to reduce false-positive predictions. A total of 3151 consensus targets were obtained for the 22 highly differentially expressed miRNAs. The GO result showed that 192 biological process categories were significantly enriched (*p* < 0.05) for these targets, including many growth-related biological processes, such as regulation of growth, cell growth, muscle cell differentiation and development, and transforming growth factor beta receptor signaling pathway.

Regulatory networks of interactions among miRNAs and their targets were constructed by using String 9.1. Many target genes in the present interaction networks have been reported to play a key role in regulation of growth. *GHR* and *GH1* are crucial genes of the hypothalamus-pituitary growth axis, which plays a major role in regulating chicken growth [47,48]. Interaction networks predicted that GHR was a target of miR-146b-3p, and this was confirmed by dual-luciferase reporter assay and qPCR in our study. Chicken miR-146b-3p may regulate muscle growth by inhibiting *GHR* expression. In human, miR-146b was reported to promote myogenic differentiation and modulate multiple gene targets in muscle cells, and might be involved in the pathogenesis of asthma and Myotonic Dystrophy Type-2 [49,50,51]. *SOCS3* and *CISH* interacted with *GHR* directly in the network analysis. As a target of miR-34c, *SOCS3* is a suppressor of cytokine signaling which affects cell proliferation, differentiation, apoptosis, and immunoregulation [52]. *SOCS3* could block insulin signaling by targeting insulin receptor substrate 1 (IRS1) and insulin receptor substrate 2 (IRS2) [53]. It also blocks the JAK/STAT pathway, relying on binding to the janus kinase. *CISH* is a target of miR-21, miR-383 and miR-205a. *CISH* is also a cytokine signaling factor, such as *SOCS3*, which was reported to inhibit GHR-JAK2 signaling to *STAT5b* by interacting with the tyrosine kinase *JAK2* or the cytoplasmic tail of *GHR* [54]. In this network, *AKT1*, another target of miR-146b-3p, was predicted to interact with *FOXO3*. The AKT-FOXO3 pathway has been reported to affect the *IGF1* pathway by regulating miR-1 expression [55]. *FOXO3* is an important member of the transcriptional regulatory factor FOXO family; it was found involved in insulin and the *IGF1* signaling pathway, which has an important role in growth [56]. The transcriptional regulatory activity of *FOXO3* can affect muscle growth, and *FOXO3* knockout mice were shown to have a stronger ability to regenerate muscle [57,58]. miR-223 and miR-142-5p were found to potentially target the *FOXO3* gene in our study. miR-223 was reported to associate with cell growth and proliferation, many signal pathways and various tumors [59,60,61]. It can regulate the expression of *IGF1R* in tumor tissue, and thus act as a potential tumor suppressor [62]. miR-223 was also found to regulate the cell proliferation by decreasing the *FOXO1* gene expression in human [63]. Some studies indicate that miR-142-5p could target and regulate cell cycle related genes, and inhibit vascular smooth muscle cell proliferation by down-regulating cell cycle progression [64,65]. The TGF-β signaling pathway is involved in many cellular processes including cell growth and differentiation [66,67], and the TGF-β signaling gene was also involved in the present network. *TGFBR2* was regulated by miR-9-3p and interacted with *TGFB* and *TGFBR1*. All these investigations indicate that the highly differentially expressed miRNAs are closely related to growth of chicken through interaction with their target genes; further studies are needed to reveal their regulatory mechanisms involved in chicken growth.

## 4. Experimental Section

### 4.1. Ethics Statement

Animal experiments were handled in compliance with and approved by the Animal Care Committee of South China Agricultural University (Guangzhou, China) with approval number SCAU#0011, 3 August 2010. All efforts were made to minimize animal subject suffering.

### 4.2. Sample Preparation

Two chicken breeds, WRR (a breed with a fast growth rate) and XH (Chinese native breed with a slow growth rate), were used for Solexa sequencing. In this study, the animals were the same as those used in our previous research on MeDIP-Seq [68]. Three female birds from each of the two-tail groups of WRR and XH at 7 weeks were selected, and then four groups including WRRh, WRRl, XHh and XHl were generated. The averages of body weight values were 1064.0 ± 11.1, 695.0 ± 24.4, 305.8 ± 23.3, and 207.6 ± 11.1 g for WRRh, WRRl, XHh, and XHl group, respectively. All chickens were euthanized by decapitation for tissues collection. Breast muscle tissues of the 12 individuals were rapidly dissected and immediately placed in liquid nitrogen then stored at −80 °C.

### 4.3. Small RNA Library Construction and Solexa Sequencing

Total RNA was extracted from each breast muscle using Trizol (Invitrogen, Carlsbad, CA, USA) according to the manufacturer’s instructions. The quality and concentration of all twelve RNA samples were determined by 1.5% agarose gel electrophoresis and absorbance at A260/280 ratio, and then divided to four mixed RNA pools for WRRh, WRRl, XHh, and XHl. Subsequently, 20 µg of total RNA were purified by denaturing 15% PAGE to enrich for 16–32 nt small RNAs, and then ligated with proprietary adapters. The ligated products were purified and reverse transcribed to cDNA to produce libraries. Finally, the libraries were deep sequenced using Genome Analyzer IIx (Illumina, San Diego, CA, USA) at Shanghai Majorbio Bio-pharm Biotechnology Co., Ltd. (Shanghai, China).

### 4.4. Sequence Analysis

The original image figure from sequencing was converted into sequence data (raw reads) by the base-calling step. For all generated raw reads, adaptor sequences, low quality reads (Sanger bases quality < 20), and contaminant reads were removed with the Fastx-Toolkit software [69]. The final data were named as clean reads. Firstly, clean reads from 18–32 nt were counted and the identical sequences eliminated. Subsequently, the assembled unique sequences were identified for the type and number of small RNA (rRNAs, tRNA, sn/snoRNAs, miRNAs, other noncoding RNAs) using Rfam databases 10.1 (http://rfam.sanger.ac.uk/). Meanwhile, the sequences were mapped to chicken genome to annotate the location in the chromosomes using Bowtie [70]. Finally, the remaining sequences were analyzed through BLASTing the miRbase 21.0 (http://www.mirbase.org/), to identify the known miRNAs in chicken. Furthermore, the novel miRNAs were predicted using miRDeep2 (https://www.mdc-berlin.de/8551903/en/) [28] from unannotated sRNAs.

### 4.5. Analysis of Differently Expressed miRNAs

DEGseq package (http://www.bioconductor.org/packages/release/bioc/html/DEGseq.html) was used to analyze differentially expressed miRNAs in this study [71]. The miRNAs expression in each library (WRRh, WRRl, XHh, and XHl) was normalized to obtain the expression of transcripts per million using total clean reads count. If the normalized reads of a given miRNAs is less than 20 in all libraries, it was removed in future differential expression analyses. Two contrasts of WRRh *vs.* WRRl and XHh *vs.* XHl were analyzed, and the fold-changes and *p*-value were calculated from the normalized reads. The differentially expressed miRNAs are identified only if |log2Ratio| > 0.585 (fold-change value > 1.5) and *p*-value < 0.05.

### 4.6. Target Gene Prediction, Pathway and Network Analysis

Both known and novel miRNAs were used to predict the miRNAs potential targets by RNAhybrid (http://bibiserv.techfak.uni-bielefeld.de/rnahybrid/) [31] and miranda (http://www.microrna.org/microrna/home.do) [30]. Only the target genes predicted by both of two methods were considered as reliable targets for further analysis. All target genes of differentially expressed miRNAs were subjected to Gene Ontology (GO) and KEGG pathway enrichment analysis by using DAVID 6.7 Functional Annotation Tool (http://david.abcc.ncifcrf.gov/). The results were filtered based on a Fisher Exact statistic methodology similar to that previously described [72]. Gene interaction analysis was performance by String 9.1 (http://www.string-db.org/) [32], and a medium Pearson Correlation Coefficient (PCC) value of 0.4 was used as a cutoff [73].

### 4.7. RNA Oligonucleotides and Transfection

The miR-146b-3p mimics, inhibitors of miR-146b-3p, mimic NC and inhibitor NC were purchased from GenePharma (GenePharma, Suzhou, China). The DF-1 chicken fibroblast cell line was cultured at 37 °C in high-glucose Dulbecco’s modified Eagle’s medium (Gibco, Grand Island, NY, USA) supplemented with 10% (*v*/*v*) fetal bovine serum (Hyclone, Logan, UT, USA), and 100 μg/mL penicillin/ streptomycin (Invitrogen). Transfection was performed with the Lipofectamine 3000 reagent (Invitrogen) combined with 50 nM of miRNA mimics, 120 nM of inhibitors, and the procedure was performed according to the manufacturer’s instructions.

### 4.8. cDNA Synthesis and Quantitative Real Time PCR (qPCR)

Total RNA of each sample and cultured cells were polyadenylated and reverse transcribed to cDNA using miScript Reverse Transcription kit (Qiagen, Valencia, CA, USA). The miScript SYBR Green PCR kit (Qiagen, Valencia, CA, USA) was used in qPCR to determine the expression of miRNA. U6 and GAPDH genes were chosen as reference genes for miRNAs and gene expression respectively. The primers of miRNAs and related genes were designed by Primer 5.0 (Table S6 in supplementary information), and universal primer of miRNAs was offered by miScript SYBR Green PCR kit. qPCR program was performed in a BIO-RAD CFX96 system (Bio-Rad Laboratories Inc., Hercules, CA, USA) as follows: 95 °C for 2 min; 40 cycles of 95 °C for 10 s, 56 °C for 30 s; 72 °C for 30 s and 72 °C for 1 min. The relative expression level of miRNA was calculated using the comparative 2^−∆*C*t^ (∆*C*t = *C*t_target gene_ − *C*t_U6_). Fold change values were calculated using the comparative 2^−∆∆*C*t^, in which ∆∆*C*t = ∆*C*t (target gene) − ∆*C*t (reference gene). All reactions were run in triplicate and presented as means ± S.E.M. The Student’s *t*-test was used to compare expression levels among different groups.

### 4.9. pmirGLO Dual-Luciferase miRNA Target Expression Vector Construction and Dual-Luciferase Reporter Assay

The sequences of *c-GHR* were amplified from the chicken genome and cloned into the pmirGLO dual-luciferase reporter vector (Promega, Madison, WI, USA) using the *Pme* I and *xho* I restriction sites. The mutant *c-GHR* plasmids were generated through changing the miR-146b-3p binding site from ATCCAT to AAGACT (Figure 7C), and mutagenesis was performed by PCR amplification and DpnI digestion to remove the parental DNA. DF-1 cells were co-transfected with 100 ng of the wild-type, mutant 3′-UTR or no-insert control dual-luciferase reporter and 50 nM of the miR-146b-3p mimic using Lipofectamine 3000 reagent in 96-well plates. After transfection for 48 h, the activities of firefly and Renilla luciferase were analysed using a dual-luciferase reporter assay system (Promega) following the manufacturer’s instructions. The luminescent signal was quantified using a Fluorescence/Multi-Detection Microplate Reader (Synergy 2, Biotek, Winooski, VT, USA) and analyzed with Gene5 software (Biotek, Bad Friedichshall, Germany).

## 5. Conclusions

In the present study, we have characterized the miRNAs expression profile of breast muscle between fast- and slow-growing chickens for the first time. There were 80 and 110 shared differentially expressed miRNAs identified in within-breed comparisons (WRRh *vs.* WRRl, and XHh *vs.* XHl) and across-breed contrasts (WRRh *vs.* XHh, and WRRl *vs.* XHl), respectively. Moreover, there were 26 miRNAs significantly differentially expressed among all four comparisons. We focused on 22 highly differentially expressed miRNAs that also were abundantly expressed. To investigate the functional roles of these miRNAs, GO analysis and the interaction network of these miRNAs and their putative targets were constructed. These integrated analyses may predict several candidates for future studies concerning miRNAs-target function on regulation of chicken growth.

## Supplementary Materials

Supplementary materials can be found at http://www.mdpi.com/1422-0067/16/07/16242/s1.
