# Supplementary material for: Deep Sequencing Analysis of miRNA Expression in Breast Muscle of Fast-Growing and Slow-Growing Broilers
Source: Int J Mol Sci. 2015 Jul 17;16(7):16242–62. doi: 10.3390/ijms160716242 (PMC4519947; doi:10.3390/ijms160716242)
Supplement: Supplementary file 1 [file ijms-16-16242-s001.pdf]

Supplementary Information

Table S1. Statistics of miRNA amounts detected from small RNA deep sequencing.

Sample	Amount of miRNAs	Total Numbers		Shared Numbers	
**WRRh**	780 (152)	891 (170)	921 (188)	680(147)	579 (102)
**WRRl**	791 (165)				
**XHh**	718 (133)	797 (165)		671 (109)	
**XHl**	750 (141)				

The value in brackets is the number of novel miRNAs. WRRh, WRRl, XHh, and XHl indicated the group of Recessive White Rock with high body weight, Recessive White Rock with low body weight, Xinhua Chickens with high body weight, and Xinhua Chickens with low body weight, respectively.

Table S2. Novel miRNAs have read counts more than 1000 reads in the libraries.

miRNA	Read Counts	Chromosome	Strand	Star	End
**15_2275**	854,769	15	−	473,792	473,870
**1_6441**	27,093	1	−	130,059,859	130,059,906
**2_9878**	6819	2	−	131,256,102	131,256,146
**3_11306**	5729	3	−	95,198,063	95,198,113
**Z_18074**	4786	Z	−	71,507,867	71,507,915
**Z_17969**	4779	Z	−	42,745,528	42,745,607
**4_12521**	1528	4	−	91,911,592	91,911,653
**4_11967**	1186	4	+	92,169,305	92,169,366
**3_11169**	1062	3	−	68,697,828	68,697,899
**5_12580**	1032	5	+	5,381,982	5,382,053

Table S3. Details of shared differentially expressed miRNAs among four contrasts.

Conjoint Contrast	Differentially Expressed miRNAs	Amount
**WRRh *vs.* WRRl& XHh *vs.* XHl**	1_4060, 1_4903, 1_5489, 10_133, 14_1786, 17_2567, 18_3584, 2_9448, 20_7112, 24_7725, 24_7726, 28_8412, 3_11092, 4_11437, 4_11488, 4_12339, 4_12341, 6_13832, 6_14144, Z_17439, Z_17672, Z_18026, miR-1416-3p, miR-146b-5p, miR-1736-5p, miR-1744-3p, miR-1751-5p, miR-1756a, miR-1791-3p, miR-205b, miR-460b-3p, miR-460b-5p, miR-6544-5p, miR-9-5p	54
**WRRh *vs.* XHh&WRRl *vs.* XHl**	1_4836, 1_5155, 1_5163, 1_5364, 1_5442, 1_5571, 1_5572, 1_6316, 1_6441, 1_6464, 1_6603, 1_6696, 1_6743, 10_168, 10_237, 11_641, 13_1554, 14_1780, 14_1987, 15_2097, 15_2173, 19_3786, 19_3829, 2_8666, 2_8901, 2_8915, 2_8923, 2_8924, 2_9621, 2_9815, 20_6874, 20_6890, 20_6954, 20_6992, 20_7045, 21_7211, 21_7222, 21_72124, 24_7682, 28_8325, 28_8457, 3_10168, 3_10670, 4_11479, 4_11670, 4_11987, 4_12193, 4_12255, 4_12363, 4_12411, 5_13090, 5_13621, 5_13622, 8_16601, Z_17715, miR-1329-5p, miR-142-5p, miR-146b-3p, miR-155, miR-1603, miR-1635, miR-1648-5p, miR-1674, 1684b-3p, miR-1716, miR-1747, miR-184, miR-194, miR-200a, miR-200b, miR-21, miR-222b-3p, miR-222b-5p, miR-223, miR-34b, miR-34c, miR-3524a, miR-3529, miR-458a-3p, miR-6557-3p, miR-6570-5p, miR-6586-5p, miR-6677-5p, miR-6681-5p	84

Table S3. *Cont.*

Conjoint Contrast	Differentially Expressed miRNAs	Amount
**All Four Contrasts**	1_6510, 15_2093, 19_3787, 23_7539, 24_7684, 26_7910, 26_8039, 26_8040, 4_11833, 4_12372, 5_12997, 5_13077, 7_15435, Z_17414，Z_18085, Z_18086, miR-122, miR-1329-3p, miR-1587, miR-1736-3p, miR-1769-3p, miR-1769-5p, miR-1773-5p, miR-205a, miR-31, miR-375	26

WRRh *vs*. WRRl indicated the comparison between the two-tail samples of Recessive White Rock; XHh *vs*. XHl indicated the comparison between the two-tail samples of Xinhua Chickens; WRRh *vs*. XHh indicated the comparison between the groups of Recessive White Rock and Xinhua Chickens with high body weight; WRRl *vs*. XHl indicated the comparison between the groups of Recessive White Rock and Xinhua Chickens with low body weight.

Table S4. Top 29 GO terms of gene counts over 1,000 for target genes of all differentially expressed miRNAs.

No.	GO Terms	GO Accession	Gene Numbers	*p* Value
1	protein binding	GO:0005515	2530	1.30×10^{-30}
2	cytoplasm	GO:0005737	1669	1.90×10^{-25}
3	cytoplasmic part	GO:0044444	1058	1.30×10^{-15}
4	cellular process	GO:0009987	3395	8.40×10^{-11}
5	membrane-bounded organelle	GO:0043227	1924	3.10×10^{-8}
6	intracellular membrane-bounded organelle	GO:0043231	1921	3.60×10^{-8}
7	binding	GO:0005488	5116	4.60×10^{-8}
8	localization	GO:0051179	1106	9.40×10^{-8}
9	cellular metabolic process	GO:0044237	2107	1.10×10^{-7}
10	intracellular part	GO:0044424	2720	1.50×10^{-7}
11	cellular macromolecule metabolic process	GO:0044260	1558	1.50×10^{-6}
12	biological regulation	GO:0065007	2107	9.10×10^{-6}
13	membrane part	GO:0044425	1635	1.20×10^{-5}
14	macromolecule metabolic process	GO:0043170	1804	1.70×10^{-5}
15	regulation of biological process	GO:0005789	2006	2.60×10^{-5}
16	intracellular	GO:0005622	3173	2.90×10^{-5}
17	nucleotide binding	GO:0000166	1171	4.00×10^{-5}
18	regulation of cellular process	GO:0050794	1922	6.20×10^{-5}
19	intrinsic to membrane	GO:0031224	1357	6.90×10^{-5}
20	metabolic process	GO:0008152	2802	1.20×10^{-4}
21	protein metabolic process	GO:0019538	1172	1.20×10^{-4}
22	integral to membrane	GO:0016021	1308	1.70×10^{-4}
23	primary metabolic process	GO:0044238	2302	1.90×10^{-4}
24	organelle	GO:0043226	2251	6.00×10^{-4}
25	intracellular organelle	GO:0043229	2247	7.00×10^{-4}
26	catalytic activity	GO:0003824	2625	1.70×10^{-3}
27	cell	GO:0005623	4723	2.00×10^{-3}
28	cell part	GO:0044464	4723	2.00×10^{-3}
29	nucleus	GO:0005634	1249	6.40×10^{-3}

Table S5. Enriched KEGG pathways for target gene of differently expressed miRNA identified in the four contrasts.

Class	ID	Pathways	*p* Value
**WRRh *vs*. WRRl**	gga04142	Lysosome	0.0023
	gga01100	Metabolic pathways	0.0061
	gga00052	Galactose metabolism	0.0078
	gga00280	Valine, leucine and isoleucine degradation	0.0078
	gga00511	Other glycan degradation	0.0093
	gga00600	Sphingolipid metabolism	0.0123
	gga00270	Cysteine and methionine metabolism	0.0137
	gga00510	N-Glycan biosynthesis	0.0151
	gga04620	Toll-like receptor signaling pathway	0.0205
	gga00230	Purine metabolism	0.0244
	gga04141	Protein processing in endoplasmic reticulum	0.0256
	gga03040	Spliceosome	0.0292
	gga04145	Phagosome	0.0327
**WRRh *vs*. XHh**	gga00052	Galactose metabolism	0.0044
	gga00280	Valine, leucine and isoleucine degradation	0.0044
	gga04744	Phototransduction	0.0053
	gga00511	Other glycan degradation	0.0053
	gga00600	Sphingolipid metabolism	0.0070
	gga01100	Metabolic pathways	0.0116
	gga04142	Lysosome	0.0127
	gga00230	Purine metabolism	0.0143
**WRRl *vs*. XHl**	gga04142	Lysosome	0.0003
	gga01100	Metabolic pathways	0.0045
	gga00052	Galactose metabolism	0.0095
	gga00280	Valine, leucine and isoleucine degradation	0.0095
	gga00511	Other glycan degradation	0.0113
	gga00600	Sphingolipid metabolism	0.0148
	gga00270	Cysteine and methionine metabolism	0.0165
	gga00510	N-Glycan biosynthesis	0.0182
	gga04620	Toll-like receptor signaling pathway	0.0245
	gga04110	Cell cycle	0.0260
	gga00230	Purine metabolism	0.0290
	gga04141	Protein processing in endoplasmic reticulum	0.0304
	gga03040	Spliceosome	0.0345
	gga04145	Phagosome	0.0384
	gga04144	Endocytosis	0.0498

Table S5. *Cont.*

Class	ID	Pathways	*p* Value
**XHh *vs*. XHl**	gga00280	Valine, leucine and isoleucine degradation	0.0002
	gga04142	Lysosome	0.0023
	gga01100	Metabolic pathways	0.0061
	gga00052	Galactose metabolism	0.0078
	gga04744	Phototransduction	0.0093
	gga00511	Other glycan degradation	0.0093
	gga00600	Sphingolipid metabolism	0.0123
	gga00270	Cysteine and methionine metabolism	0.0137
	gga04270	Vascular smooth muscle contraction	0.0218
	gga04110	Cell cycle	0.0218
	gga04145	Phagosome	0.0327
	gga04530	Tight junction	0.0449

WRRh *vs*. WRRl indicated the comparison between the two-tail samples of Recessive White Rock; XHh *vs*. XHl indicated the comparison between the two-tail samples of Xinhua Chickens; WRRh *vs*. XHh indicated the comparison between the groups of Recessive White Rock and Xinhua Chickens with high body weight; WRRl *vs*. XHl indicated the comparison between the groups of Recessive White Rock and Xinhua Chickens with low body weight.

Table S6. Detail of miRNA quantitative real-time PCR primer.

Gene	Primer Sequence (5′ to 3′)	Annealing Temp (°C)
**gga-miR-223**	F: GTGTCAGTTTGTCAAATACCCCAA	56
**gga-miR-142-5p**	F: GCCCATAAAGTAGAAAGCACTACAA	56
**gga-miR-205a**	F: CCTTCATTCCACCGGAGTCTG	56
**gga-miR-222b-5p**	F: GCTCAGTAGTCAGTGTAGGATCTG	56
**U6**	F: GCAGGGCCATGCTAATCTTCTCTGTATCG	56
**GHR**	F: TTCAACACATCCTACACCTCG R: TGGTGGTGGATCCCATCGTA	60
**GAPDH**	F: TATGATTCTACACACGGAC R: ATGGTGGTGAAGACACCAGTG	60

F means forward primer; R means reverse primer.

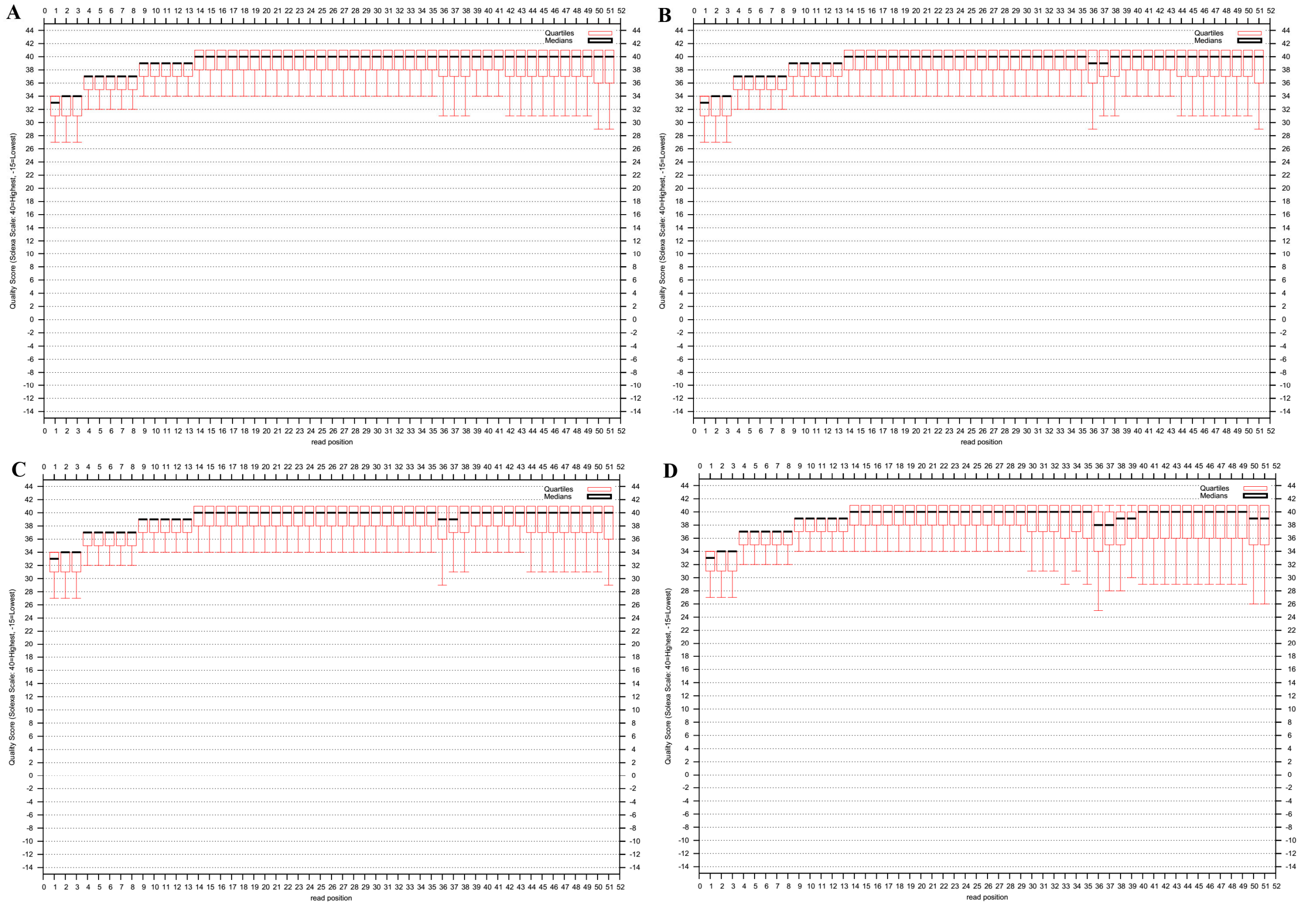

Figure S1. The quality data for RNA samples. (**A**) WRRh (Recessive White Rock with high body weight); (**B**) WRRl (Recessive White Rock with low body weight); (**C**) XHh (Xinhua Chickens with high body weight); (**D**) XHl (Xinhua Chickens with low body weight). Horizontal axis means base of reads 1–51 from 5′ to 3′; vertical axis means quality value of base. Red boxes means quartiles of reads quality, and black bold lines means medians of reads quality. The higher the medians, the lower the error rate of sequencing.

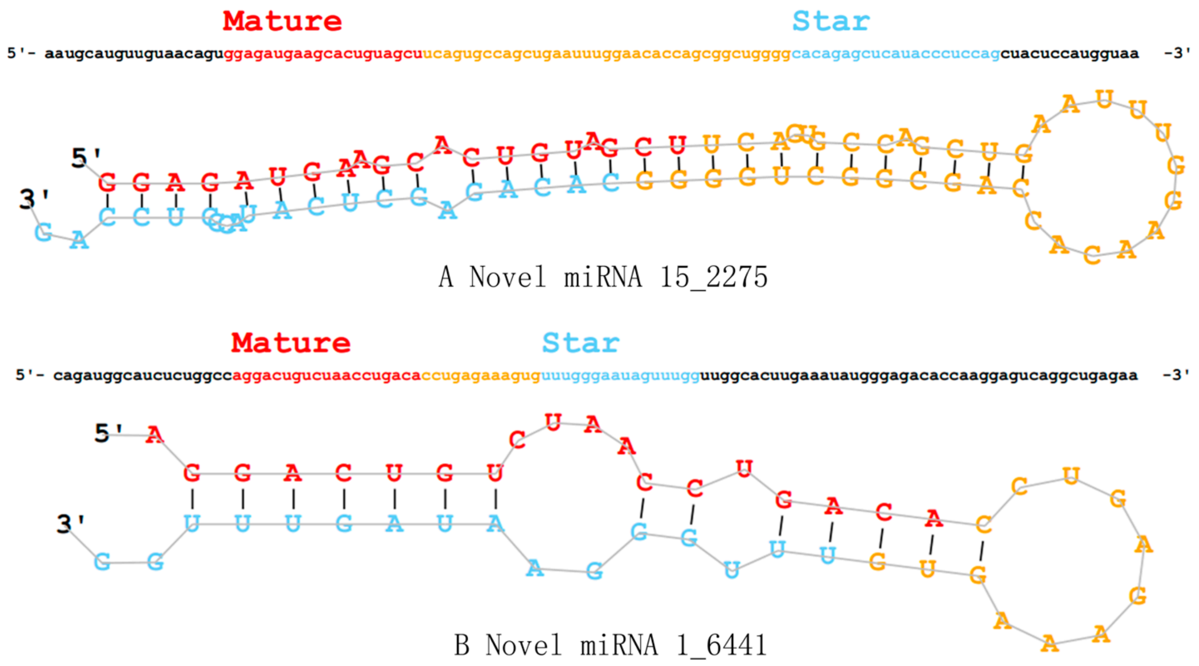

Figure S2. Predicted secondary structures of two most abundant novel miRNAs. The red color indicated the mature sequence, the yellow color indicated the loop sequence, and the blue color indicated the predicted star sequence.

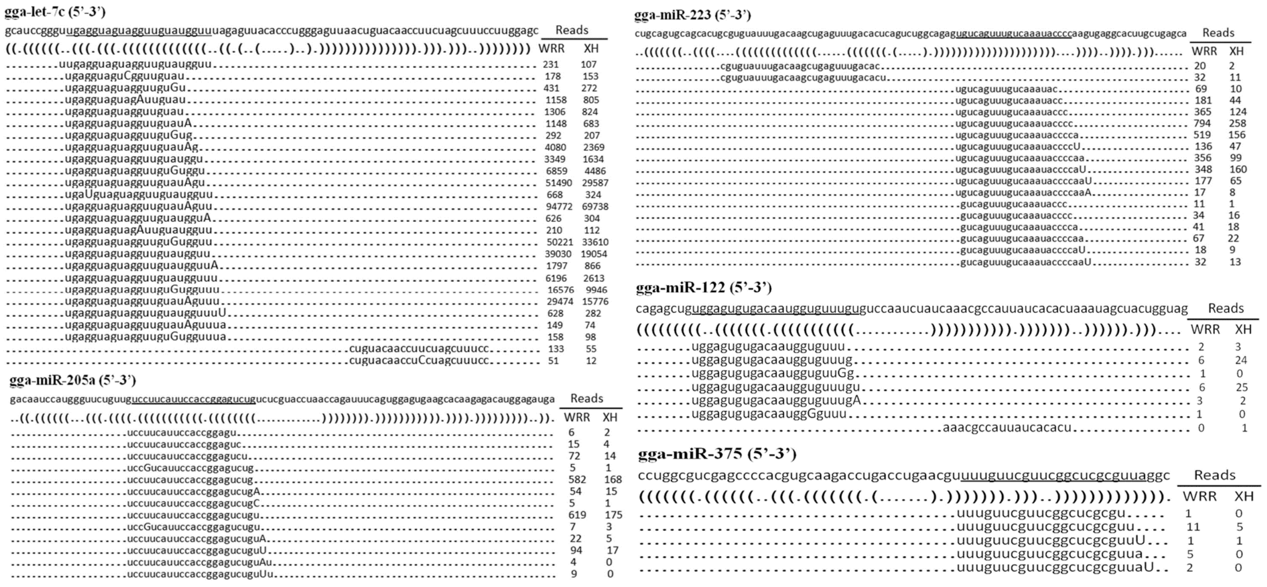

Figure S3. Various isomiRs of five gga-miRs. Reads alignments of the various isoforms of five gga-miRs are presented. The sequence of the gga-miR hairpin is presented in the top line, and underline letter means mature sequence; the brackets below denote the secondary structure. Reads that aligned with the mature gga-miR sequence are denoted by a series of dots and the mutations are denoted by uppercase. The number of reads for each sequence is presented on the right.
